# A New Rectus and Sartorius Sparing Approach for Periacetabular Osteotomy in Patients with Developmental Dysplasia of the Hip

**DOI:** 10.3390/jcm10040601

**Published:** 2021-02-05

**Authors:** Jannis Löchel, Viktor Janz, Carsten Perka, Andre Hofer, Alexander Zimmerer, Georgi I. Wassilew

**Affiliations:** 1 Center for Musculoskeletal Surgery, Charité—Universitätsmedizin Berlin, Corporate Member of Freie Universität Berlin, Humboldt-Universität zu Berlin, and Berlin Institute of Health, Augustenburger Platz 1, D-13353 Berlin, Germany; carsten.perka@charite.de; 2 Department for Orthopaedic Surgery, University of Greifswald, Ferdinand-Sauerbruch-Straße, D-17475 Greifswald, Germany; viktor.janz@med.uni-greifswald.de (V.J.); andre.hofer@med.uni-greifswald.de (A.H.); alexander.zimmerer@gmail.com (A.Z.); georgi.wassilew@med.uni-greifswald.de (G.I.W.)

**Keywords:** periacetabular osteotomy, approach, muscle sparing, rectus sparing, sartorius sparing

## Abstract

Background: periacetabular osteotomy (PAO) is known as the gold standard surgical treatment in young adults with symptomatic hip dysplasia. With the aim of reducing soft tissue trauma, we developed a new rectus and sartorius sparing (RASS) approach. We hypothesized that this new PAO technique was equal regarding acetabular reorientation, complication rate, and short-term clinical outcome parameters, compared to our conventional, rectus sparing (RS) approach. Patients and Methods: we retrospectively assessed all PAO procedures performed by a single surgeon between 2016 and 2019 (n = 239 hips in 217 patients). The cases in which the new RASS technique were used (n = 48) were compared to the RS cases for acetabular orientation parameters, surgical time, perioperative reduction of hemoglobin level, and length of hospital stay (LOHS). Inclusion criteria were a lateral center-edge angle (LCEA) <25° and osteoarthritis Tönnis grade ≤1. Patients with acetabular retroversion or additional femoral osteotomy were excluded. Results: the mean patient age at the time of surgery was 29 years (14 to 50, SD ± 8.5). Females accounted for 79.5% in this series. The mean preoperative LCEA were 16° (7 to 24°, SD ± 4.4) and 15° (0 to 23°, SD ± 6) in the RASS and the RS group, respectively (*p* = 0.96). The mean preoperative acetabular index (AI) angles were 14° (2 to 25°, SD ± 4) and 14° (7 to 29°, SD ± 4.3), respectively (*p* = 0.67). The mean postoperative LCEA were significantly improved to 31° (25 to 37°, SD ± 3.5, *p* < 0.001) and 30.2° (20 to 38°, SD ± 4, *p* < 0.001), respectively. The mean postoperative AI angles improved to 2.8° (−3 to 13°, SD ± 3.3, *p* < 0.001) and 3° (−2 to 15°, SD ± 3.3, *p* < 0.001), respectively. There were no significant differences between the RASS and the RS group for surgical time, perioperative reduction in hemoglobin level, and LOHS. No blood transfusions were necessary perioperatively in either group. No major perioperative complication occurred in either group. We observed one surgical site infection (SSI) requiring superficial debridement in the RS group. Conclusion: the RASS approach for PAO showed to be a safe procedure with equivalent acetabular reorientation and equivalent clinical outcome parameters compared to the RS approach. Additionally, patients have fewer postoperative restrictions in mobilization with the RASS approach.

## 1. Introduction

Developmental dysplasia of the hip (DDH) has been identified as the most frequent cause for early, secondary osteoarthritis in young patients [1,2]. In 1988, Ganz et al. published a surgical, joint preserving technique for acetabular reorientation in patients with symptomatic DDH [3]. This technique, known as Bernese periacetabular osteotomy (PAO), allows to optimize the acetabular coverage of the femoral head and thereby improve the biomechanical loading of the joint, showing overall good long-term results [4,5]. Reorientation of the acetabular fragment in an optimal position remains the most challenging surgical step. The conventional surgical technique requires considerable exposure and is in the immediate vicinity of multiple neurovascular structures. Even in the hands of experienced surgeons, significant complications have been reported in the literature [3,6,7,8].

Reducing the soft tissue trauma, maintaining adequate exposure, and preventing severe complications remain the goal when preforming PAO. With the above-mentioned goals, we developed a new rectus and sartorius sparing (RASS) approach for PAO.

Since the initial description of the PAO technique, different approaches have been reported [2]. The Smith-Petersen approach is the most commonly used approach [6,9,10]. Less invasive techniques, such as rectus tendon sparing and transsartorial approaches are reported to minimize surgical trauma, reduce surgical time, blood loss, and length of hospital stay (LOHS) [11,12,13,14].

For the transsartorial approach, which is currently the PAO technique offering the least exposure, the sartorius muscle is split in fiber direction. Using our RASS technique, the ischium and pubis osteotomy are performed through the interval between the iliopsoas and rectus muscle after medialization of the sartorius and lateralization of the tensor fasciae latae muscle. The muscle insertions and muscle bellies remain intact. To our knowledge, this technique is currently the one with the fewest exposure.

Our new RASS approach may affect the acetabular reorientation and the perioperative complication rate, compared to our standard, RS approach. We hypothesized that (1) acetabular reorientation, (2) perioperative complication rate, and (3) clinical outcome parameters were equivalent using the RASS compared to the RS technique.

## 2. Patients and Methods

Between 2016 and 2019, 239 consecutive PAOs (in 217 patients) were performed by the senior author at two consecutive departments (department A from 2016 until 2018, department B in 2019). Patients were identified using our institutional database, the data work-up was performed according to the Strengthening the Reporting of Observational Studies in Epidemiology (STROBE) checklist [15]. Institutional review board approvals were obtained prior to the initiation of this study. PAOs were performed in symptomatic patients with: a lateral center-edge angle (LCEA) <25°, a congruent joint space, osteoarthritis Tönnis grade ≤1. Patients with acetabular retroversion or additional femoral osteotomy were excluded. In 111 cases (46.4%) of reduced femoral offset and an intraoperative internal rotation capacity <30° in 90° of hip flexion, a simultaneous femoral head-neck offset correction was performed through the interval between tensor and sartorius muscle until 30° of internal rotation were achieved.

The senior authors standard, RS PAO approach consisted of a rectus sparing PAO with detachment of the sartorius muscle insertion from the anterior superior iliac spine (ASIS) via an osseous wafer and transosseous reattachment with non-absorbable sutures during wound closure [16]. A total of 191 procedures were performed using the conventional technique, and in 48 cases, the RASS technique was used. A femoral head-neck offset correction was performed in 22 patients (46%) in the RASS group and 89 patients (47%) in the conventional group.

The mean patient age at the time of surgery was 29 years (14 to 50, SD ± 8.5); 190 were female (79.5%) and 49 were male (20.5%). The mean body mass index (BMI) was 28 kg/m^2^ (18 to 42 kg/m^2^, SD ± 6.2). Clinical and radiological follow-up were obtained upon discharge and at six and 12 weeks, postoperatively. The pre- and postoperative radiographs were reviewed by two of the authors (VJ and JL).

### 2.1. Preoperative Diagnostics

Radiologic diagnostics include an anterior-posterior (AP) radiograph of the pelvis in a standing position to examine the joint position under loading conditions. A Dunn view and an MRI with radial sequences were also performed.

### 2.2. Anesthesia and Patient Positioning

All PAOs were performed in general anesthesia and under hypotension. A transversus abdominis plane block was performed directly preoperatively as well as a continuous intraoperative infusion of tranexamic acid (TXA) [17,18]. Patients were placed in a supine position on a fully radiolucent table, with draping allowing free motion of the ipsilateral leg.

### 2.3. Surgical Technique

**Figure 1:**
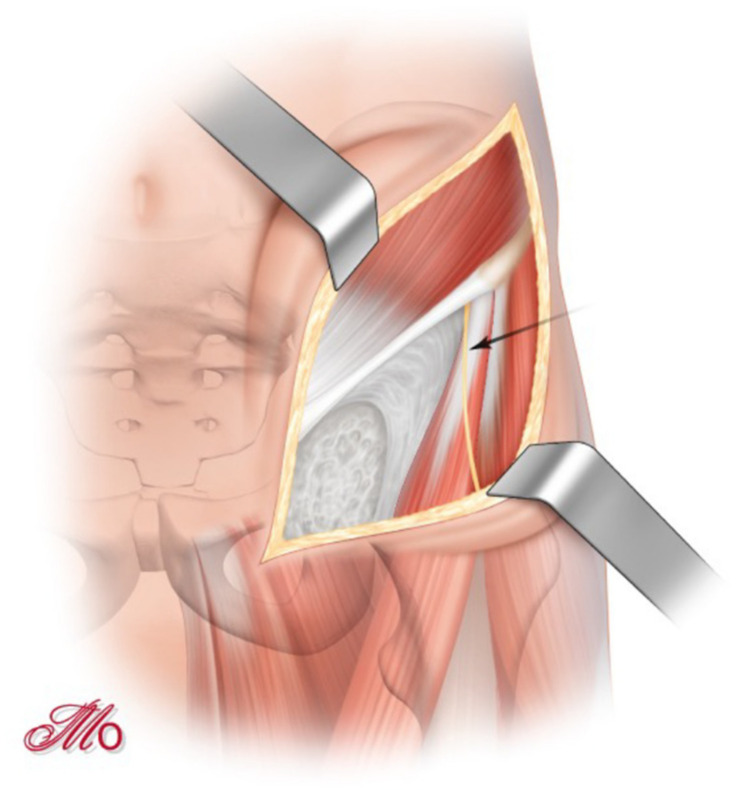
‘Bikini-shaped’ skin incision and identification of the lateral femoral cutaneous nerve (arrow-marked).

The skin incision begins two centimeters lateral of the anterior third of the iliac crest and is extended medially and caudally of the ASIS and continued approximately one centimeter proximal and parallel to the inguinal fold in the direction of the pubic symphysis (SP) (Figure 1). During the subcutaneous dissection, the lateral femoral cutaneous nerve (LFCN) is identified and preserved whenever possible.

**Figure 2:**
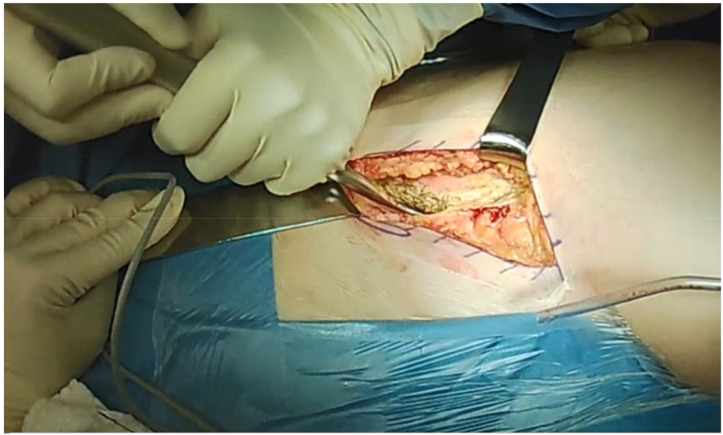
Careful detachment of the abdominal muscles and opening the first ilioinguinal window.

Two surgical windows are used to perform the osteotomies. The hip is flexed to relax the anterior soft tissue and neurovascular structures and to facilitate soft tissue management. To open the first ilioinguinal window, a careful detachment of the abdominal muscles and the iliacus muscle, as well as a partial detachment of the inguinal ligament, are performed (Figure 2).

**Figure 3:**
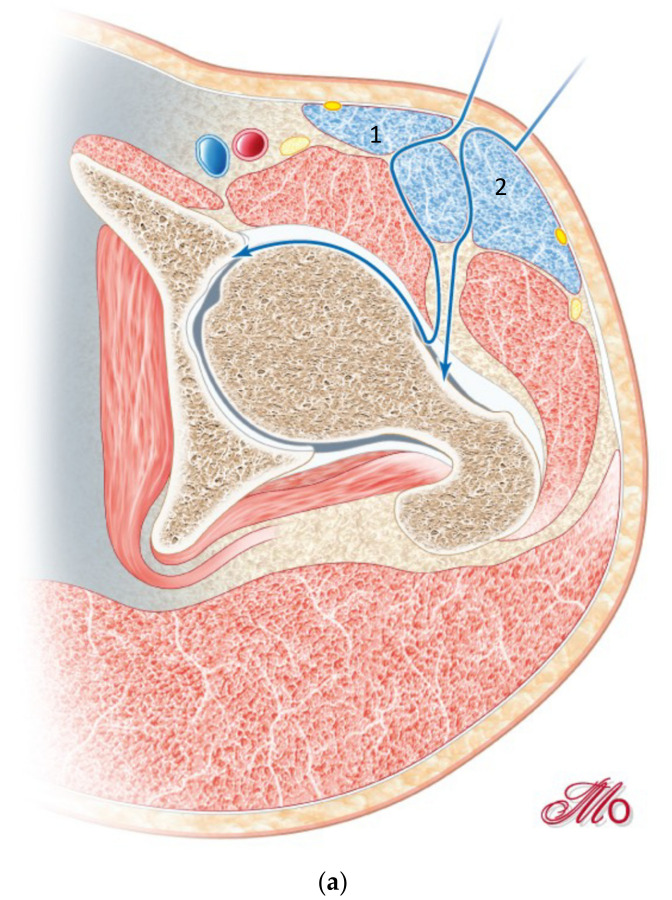

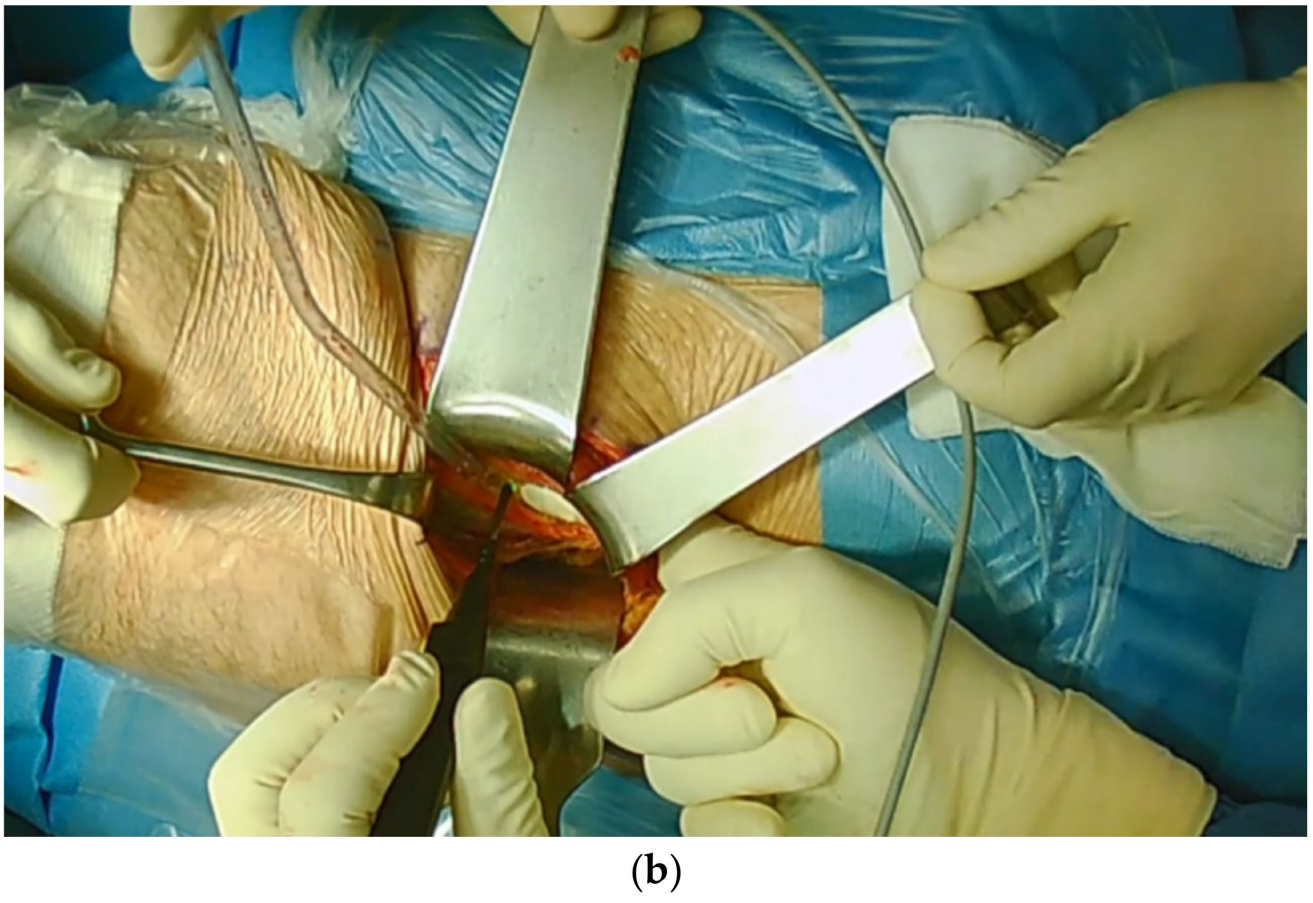
(**a**) Opening the second window, by retracting the sartorius muscle (1) medially and the tensor muscle (2) laterally. (**b**) Exposure of the rectus fascia through the first window.

To open the second window, the sartorius muscle is retracted medially, the tensor muscle and LFCN laterally, exposing the rectus fascia (Figure 3). The rectus fascia can be bluntly mobilized through the first window and is split in fiber direction through the second window.

After dissection of the fascia, fibers of the iliocapsularis muscle from the anterior inferior iliac spine (AIIS) and the rectus muscle are exposed. The interval between these muscles is dissected at the AIIS and extended distally. The iliopsoas is retracted medially using a pointed Hohmann retractor placed onto the superior pubic ramus and the rectus muscle is medialized, partially exposing the capsule. Through this interval, a Cobb elevator is inserted to bluntly dissect the tissue attached to capsule, exposing the site of the first osteotomy. The capsule should not be perforated as this may subsequently misdirect the osteotome in the intraarticular space.

The incomplete ischium osteotomy begins at the infracotyloid groove, which can be safely identified with the curved osteotome and is performed in an ascending fashion towards the ischial spine. The ischium osteotomy should be performed using fluoroscopy to prevent intraarticular penetration and posterior column transection. The osteotomy is initiated medially and should end 1 cm before the ischial spine but at latest with the opposite cortex. Hip flexion, slight abduction, and external rotation may facilitate the medial ischium osteotomy by relaxing the soft tissue. The osteotomy is then continued laterally. Hip extension, abduction, and external rotation relax and distance the ischial nerve. The lateral portion of the ischium is thinner than the medial portion and the osteotomy of the lateral portion is therefore shorter than medial. The osteotomy of the lateral portion should be performed carefully and end before the opposite cortex to prevent an ischial nerve injury. Orienting the osteotome towards the contralateral shoulder can help to avoid a lateral perforation of the ischium.

The second osteotomy is the pubic osteotomy. Adequate exposure of the pubis is achieved using a pointed Hohmann retractor placed onto the superior pubic ramus. After longitudinal incision of the periosteum, two blunt retractors can be placed within the periosteal flaps to protect the adjacent obturator nerve and vessel. Preservation of the periosteal sleeve also helps to maintain vascularity and decreases the risk of pseudarthrosis formation. The osteotomy is localized approximately one centimeter medial of the iliopectineal eminence and is initiated using a saw and completed by an osteotome.

The third osteotomy is the supraacetabular ilium osteotomy. The exposure of the outer part of the ilium is achieved with limited soft tissue dissection, to protect the abductors and the inferior branch of the superior gluteal artery, responsible for acetabular blood supply. The supraacetabular osteotomy is performed using a saw and initiated distal to the ASIS to preserve the origin of the sartorius muscle. In case of a very distal ASIS, the osteotomy is initiated in an ascending direction before the horizontal part is performed. The osteotomy ends approximately one centimeter before the sciatic notch. This ensures for adequate bone stock and preservation of the acetabular vascular supply.

The fourth osteotomy is the retroacetabular osteotomy. Using fluoroscopy, the retroacetabular space can be visualized and a Kirschner wire (K-wire) may be placed exactly in the middle of the posterior column in line with the upcoming retroacetabular osteotomy. The K-wire can be used as guidance for the osteotome to prevent an intraarticular osteotomy or a fracture of the posterior column. Exposure is achieved by inserting the tip of a blunt retractor on the inner cortex of the sciatic notch. The retroacetabular osteotomy is initiated at the medial end of the supraacetabular osteotomy and is directed towards the end of the ischium osteotomy. An angled osteotome is used to break any residual bone in the distal portion of the retroacetabular osteotomy at the junction to the ischial osteotomy. The acetabular bone at this junction is thicker than in the more superior part of the posterior acetabular column and this step helps to complete the retroacetabular osteotomy from medial to lateral, if necessary.

**Figure 4:**
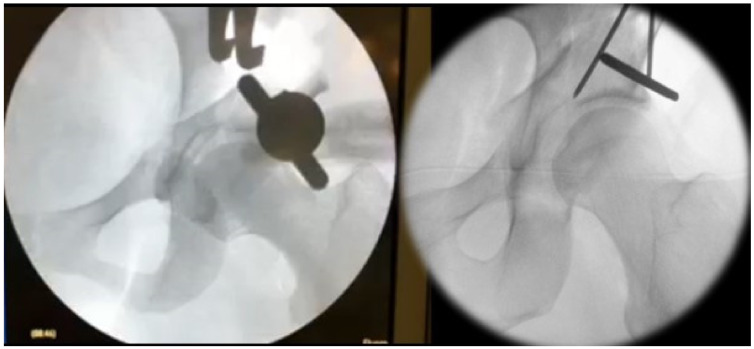
Mobilization of the acetabular fragment using a Schanz screw and a distractor followed by preliminary K-wire fixation.

To further mobilize the acetabular fragment one or two 5 mm Schanz screws are placed in the supraacetabular bone stock and a distractor is placed at the junction of the supra- and retroacetabular osteotomy (Figure 4). All acetabular osteotomies are now complete. However, acetabular mobility can still be impeded by periosteum or the surrounding soft tissue. Through simultaneous manipulation of the Schanz screw and distractor, adequate mobilization of the acetabular fragment can be achieved. The acetabular fragment can now be reoriented according to the underlying pathology. Commonly, we aim for a LCEA of 30° with an ascending acetabular slope and an anteverted acetabulum. For preliminary fixation of the acetabulum two or three 2.5 mm K-wires are used.

After a detailed fluoroscopic assessment of the final acetabular reorientation, definite fixation is achieved by replacing the K-wires with 4.5 mm cortical screws.

The range of motion of the hip (ROM) is intraoperatively examined and should allow 120° of flexion and 30° of internal rotation in 90° hip flexion. If the ROM testing reveals a secondary femoroacetabular impingement (FAI), despite an optimal acetabular reorientation, an arthrotomy, and head–neck offset correction are performed over the existing Smith–Peterson approach using a high-speed burr.

### 2.4. Postoperative Treatment

Both study groups were allowed immediate postoperative partial weight-bearing of 15 kg for four weeks and transitioned into full weight bearing thereafter. Patients with extensive reorientation and resulting large osteotomy gaps followed six weeks of 15 kg partial weight-bearing. For the RS group active hip flexion was prohibited for six weeks to reduce the tension of the sartorius muscle on the reattached ASIS. Patients of the RASS group were allowed free active hip flexion. Patients, in which an arthrotomy and additional head–neck offset correction were performed, received an extended course of heterotopic ossification prophylaxis with Etoricoxib 90 mg once daily for 21 days.

## 3. Statistical Analysis

The mean values and ranges were calculated for demographic data. Categorical variables were described with percentages. The Wilcoxon test was used to test for statistical significance for nonparametric data within one group. The chi-square test was used for comparison of categorical variables between both groups. The Mann–Whitney U test was used for nonparametric data. Statistical significance was defined as *p* < 0.05. All statistical analyses were conducted with SPSS 27 (IBM Corp., Armonk, New York, NY, USA).

## 4. Results

### 4.1. Radiographic Outcome

The mean preoperative lateral center-edge angle (LCEA) were 16° (7 to 24°, SD ± 4.4) and 15° (0 to 23°, SD ± 6) in the RASS and the RS group, respectively (*p* = 0.96). The mean preoperative acetabular index (AI) angles were 14° (2 to 25°, SD ± 4) and 14° (7 to 29°, SD ± 4.3), respectively (*p* = 0.67). The mean postoperative LCEA were significantly improved to 31° (25 to 37°, SD ± 3.5, *p* < 0.001) and 30.2° (20 to 38°, SD ± 4, *p* < 0.001), respectively. The difference between the postoperative LCEA angles of both groups was not significant (*p* = 0.3). The mean postoperative AI angles improved to 2.8° (−3 to 13°, SD ± 3.3, *p* < 0.001) and 3° (−2 to 15°, SD ± 3.3, *p* < 0.001), respectively. The difference between the postoperative AI angles of both groups was not significant (*p* = 0.9). Five cases (10%) of the RASS and 21 cases (11%) of the RS group had postoperative overcorrection, in two cases of the RASS group with a postoperative acetabular retroversion. We observed no postoperative loss of reorientation in either group. The target zone of LCEA angles reached 39/48 cases (81%) in the RASS and 157/191 cases (82%) in the RS group, respectively.

### 4.2. Clinical Outcome

No delayed or non-union was observed in either group. No blood transfusions were necessary perioperatively in either group. We observed one postoperative surgical site infection in the RS group. LFCN injury and subsequent lateral thigh paresthesia was observed in 24 patients (50%) of the RASS and 109 patients (57%) of the RS group (*p* = 0.4).

The mean duration of surgery was 86 min (57 to 142, SD ± 25) in the RASS group and 87 min (50 to 168, SD ± 25) in the conventional group (*p* = 0.8). The mean LOHS was 8.3 (6 to 14, SD ± 1.7) and 8.5 days (5 to 17, SD ± 1.6), respectively (*p* = 0.85). The mean preoperative hemoglobin level was 8.9 mmol/l (7.5 to 10.2, SD ± 0.89) and 8.5 mmol/l (7.1 to 10.3, SD ± 0.72) in the RASS and the RS group, respectively (*p* = 0.12). The mean postoperative reduction in hemoglobin level was 1.9 mmol/l (0.3 to 3 mmol/l, SD ± 0.7) and 1.7 mmol/l (0.7 to 3.7 mmol/l, SD ± 0.0.64) in the RASS and the RS group, respectively (both *p* < 0.001). The postoperative reduction in hemoglobin comparing RASS and RS group was not significant (*p* = 0.2).

## 5. Discussion

The main finding of this study is that acetabular reorientation can be reliably and safely achieved via a RASS approach, in the same manner as via a RS approach [14,19]. The study is the first to compare the radiological and clinical outcome of PAOs performed via a RASS and RS approach. The target zone of postoperative LCEA angles was reached in a high proportion of cases in both groups, showing that the acetabular reorientation was not affected by the more limited exposure of the RASS approach. The pre- and postoperative LCEA and AI angles are comparable with previously published studies investigating PAO approaches with reduced exposure [14,20].

Patients undergoing RASS PAO benefit from fewer postoperative mobilization restrictions as they are allowed free active hip flexion. This could have an impact on long-term hip flexion capability, being investigated in subsequent studies.

No major perioperative complications occurred in either group; therefore, we consider the RASS PAO technique to be a safe procedure. However, to minimize the risk of osteotomy-associated complications, such as intraarticular fractures or extension of the osteotomies through the posterior column, fluoroscopic control is recommended while performing the osteotomies.

All secondary outcome parameters (surgical time, perioperative reduction in hemoglobin level and LOHS) of the RASS PAO group were equivalent to those of the RS PAO group and in accordance with the current literature [14,19,21]. The reduction in surgical time, through a quicker exposure and the omitted refixation of the sartorius muscle, in the RASS PAO group, may be offset by a more challenging soft tissue management while performing the osteotomies. The perioperative blood loss from the soft tissue is insignificant; the reduction in hemoglobin level in both groups is associated with bleeding from the osteotomy surfaces. Additionally, overall blood loss is dependent on the use of TXA and overall surgical time [18]. The mean LOHS in the RASS PAO group did not differ compared to the RS group and cannot be utilized as a surrogate parameter for early functional outcome. This study was performed in Germany and the minimum LOHS after PAO in the German health care system is six days. An earlier discharge is associated with reimbursement penalties for the orthopedic department. Therefore, it is not possible to draw any conclusions about a suspected improved early functional outcome or possible earlier hospital discharge through an RASS approach from our study design, due to systemic structural bias.

A satisfactory postoperative functional outcome is further dependent on free hip flexion and adequate internal rotation ability. Despite an optimal acetabular reorientation, PAO can result in secondary FAI, due to a reduced femoral offset. A critical, intraoperative assessment of the flexion and internal rotation of the hip joint must be performed in every case. Some authors believe less invasive approaches for PAO to be unsuitable to perform a simultaneous arthrotomy for addressing femoral neck pathologies [14,22]. Our described technique for RASS PAO allows for easy arthrotomy, to perform femoral head-neck offset correction via the used Smith–Peterson or Hueter approach, which has been described primarily in 1882 [23]. We recommend arthrotomy and simultaneous femoral head-neck offset correction when hip flexion and internal rotation are reduced after acetabular reorientation.

Intraoperative LFCN injury can occur by direct and indirect, pressure caused damage. The range of LFCN injury in the literature is considerable [9]. Some authors identified risk factors for higher LFCN injury rates such as ASIS osteotomy [24]. Recent studies differentiated the LFCN branching in three types with nearly equal prevalence. The fibers of the ‘fan-type’, characterized by multiple thin branches, cannot be protected effectively during PAO [25,26]. We observed a postoperative LFCN dysesthesia rate of 50% and 57% in the RASS and the RS group, respectively. This compares higher to previously published research [9,14]. Troelsen et al. published no postoperative LFCN dysesthesia. These results should be interpreted cautiously as we know about variable LFCN courses.

One limitation of this study is that the indication for performing a RASS and RS PAO was not randomized. A further limitation is the lack of objective and subjective functional outcome parameters. Additional follow-up studies are necessary, to detect possible differences in the long-term joint survival and outcome of RASS and conventional PAO patients.

In conclusion, we were able to show that the novel RASS approach for PAO is a safe procedure, which allows a free reorientation of the acetabulum and shows comparable complication rates and equivalent outcome to RS PAO.

## Data Availability

The data presented in this study are available on request from the corresponding author.
